# Immunization with CENP-C Causes Aberrant Chromosome Segregation during Oocyte Meiosis in Mice

**DOI:** 10.1155/2021/4610494

**Published:** 2021-01-30

**Authors:** Jiao Fan, Yang Liu, Yiping Zhong

**Affiliations:** ^1^The Obstetrics and Gynecology Hospital Affiliated to Fudan University, No. 419 of Fangxie Road, Shanghai City, China; ^2^Reproductive Medicine Center for the First Affiliated Hospital of Sun Yat-sen University, No. 58 of Zhongshan Road II, Guangzhou City, Guangdong Province, China

## Abstract

Anticentromere antibodies (ACA) were associated with lower oocyte maturation rates and cleavage rates, while the mechanism was not clear. Aims of this study were to examine whether active immunization with centromere protein C could elicit the CENP-C autoantibody in mice and the impacts of the CENP-C autoantibody on oocyte meiosis. Mice were divided into two groups, one was the experimental group immunized with human centromere protein C and Freund's adjuvant (CFA), and the other was the control group injected with CFA only. Serum and oocytes of BALB/c mice immunized with human centromere protein C (CENP-C) in complete Freund's adjuvant (CFA) or injected with only CFA were studied for the development of the CENP-C antibody. Rates of germinal vesicle breakdown (GVBD), first polar body (Pb1) extrusion, abnormal spindle morphology, and chromosome misalignment were compared between the experimental group and the control group. The CENP-C antibody was only observed in serum and oocytes of mice immunized with the centromere protein C antigen. The first polar body (Pb1) extrusion rate was lower in the experimental group (*P* < 0.01). A higher percentage of spindle defects and chromosome congression failure were also detected in the experimental group (spindle defects: 64.67 ± 1.16% vs. 9.27 ± 2.28% control; chromosome misalignment: 50.80 ± 2.40% vs. 8.30 ± 1.16% control; *P* < 0.01 for both). Oocyte meiosis was severely impaired by the CENP-C antibody, which may be the main mechanism of adverse reproductive outcomes for ACA-positive women who have no clinical symptoms of any autoimmune diseases.

## 1. Introduction

Antinuclear antibodies (ANA) were related to infertility, decline of oocyte quality, impairment of embryo development, recurrent spontaneous abortion, IVF failure [1–5]. The pregnancy rate and implantation rate in women with positive ANA were lower than those in women with negative ANA. ANA were a large group of autoantibodies targeting the entire cell, including DNA, RNA, proteins, and/or their complexes. It is unknown which specific kinds of ANA were involved in poor reproductive outcomes. It has been reported that the anticentromere antibody (ACA), a kind of ANA, may be an essential marker for flawed oocytes in infertile women with any type of ANA. Our previous study [1] and Shirota et al.'s study [6] revealed patients who were positive for anticentromere antibodies (ACA) had a lower percentage of mature oocytes and a lower rate of embryo cleavage than women negative for ACA. These results indicated ACA had adverse impacts on oocyte maturation and embryo development. But the mechanism of adverse reproductive outcomes caused by ACA was not clear.

Centromere proteins (CENP), a special region in heterogeneous chromosomes and presented morphologically as primary constriction in chromosomes, are composed of a number of conserved protein complexes, including CENP-A, CENP-B, CENP-C, CENP-D, and CENP-T [7–9]. A kinetochore, a large protein complex assembled on the centromeric heterochromatin regions of the chromosomes, could be divided into three parts in cell metaphase: inner kinetochore, central kinetochore, and outer kinetochore [10]. The inner kinetochore contains centromere protein A (CENP-A), centromere protein C (CENP-C), centromere protein T (CENP-T), and other centromere proteins. The central domain is the region between the outer and inner kinetochores. The outer kinetochore, composed of a number of protein complexes such as Knl1, Mis12, and Ndc80 complexes, is required for stable kinetochore-microtubule (KT-MT) attachment and recruitment of the spindle assembly checkpoint (SAC) [10]. SAC could prevent anaphase onset until all chromosomes are stably attached to microtubules and accurately aligned in the equatorial plate in cell metaphase. Stable kinetochore-microtubule attachment and correct chromosome alignment in cell metaphase are responsible for producing haploid gametes from parental cells. The molecular structure is the basis of molecular function. Not only are structures of centromere proteins and kinetochore proteins interdependent, but also functions of centromere proteins and kinetochore proteins interplay. Some molecules such as CENP-A, CENP-C, and CENP-T, which served as inner kinetochore construction, were protein components of centromeres. The N-terminal of CENP-C binds to Mis12, which suggests that CENP-C links the centromeric chromatin with the outer kinetochore [11]. Therefore, CENP-C, a component of the inner kinetochore, appears to bridge the inner kinetochore and outer kinetochore and is essential for cell mitosis. The aim of the study was to investigate the effects of the CENP-C antibody produced by the autoimmune method in mice on oocyte meiosis.

## 2. Materials and Methods

### 2.1. Animals

All mice used in this study were 6- to 7-week-old BALB/c female mice purchased from the laboratory animal center of the east campus of Sun Yat-sen University. Animals were maintained with food and water under a 14-hour light/10-hour dark cycle in the laboratory animal center of the north campus of Sun Yat-sen University. All procedures for mouse care and use were conducted in accordance with the guidelines and approved by the Institutional Animal Care and Use Committees of Sun Yat-sen University. A total of 24 mice were used in our experiments.

### 2.2. Active Immunization with Centromere Protein C

Mice were divided into two groups according to treatment, one was the experimental group immunized with human CENP-C protein plus complete Freund's adjuvant, and the other was the control group injected with complete Freund's adjuvant only. The first immunization was as follows: human CENP-C (50 *μ*g) (Proteintech, Chicago, USA) was emulsified in complete Freund's adjuvant (CFA) and injected subcutaneously into the back of mice in the experimental group. Mice in the control group were injected with CFA alone. Two weeks later, all mice received the boost immunization: Human CENP-C (25 *μ*g) (Proteintech, Chicago, USA) was emulsified in complete Freund's adjuvant (CFA) and injected subcutaneously into the back of mice in the experimental group. Mice in the control group were injected with CFA alone. The volume of reagents given for each mouse was the same each time. Ovulation induction for all mice was started 7 days after the second immunization or injection. Mice were bled on the oocyte pick-up day to detect the development of the serum CENP-C antibody and killed.

### 2.3. Serum CENP-C Antibody Detection

#### 2.3.1. ELISA

Mouse blood was collected at the ovum pick-up day and centrifuged to discard the particulate materials on the bottom. Mouse serum was collected. The amount of the anti-CENP-C antibody in the serum was quantified using the Anti-Centromeres ELISA (IgG) kit (EUROIMMUN Medizinische Labordiagnostika AG, Germany) following the manufacturer's instructions, and the absorbance value was detected at the 450 nm filter. We changed the HRP-conjugated anti-human secondary antibody in the kit to the HRP-conjugated anti-mouse secondary antibody (GB23301, Google Biology, China). All samples were evaluated in duplicate.

### 2.4. GV Oocyte and MII Oocyte Collection

Germinal vesicle (GV) oocytes were retrieved from female BALB/c mice 48 hours after intraperitoneal injection of 10 IU pregnant mare serum gonadotropin (PMSG) into mice. To obtain fully grown GV oocytes, mouse ovaries were cut up, and then GV oocytes would release from ovaries. For *in vitro* maturation, GV oocytes were cultured in the M2 medium under mineral oil at 37°C in a 5% CO2 incubator. Three hours later, we detected and recorded amounts of oocytes transformed into the GVBD stage in order to achieve information about the impact of the anti-CENP-C antibody on oocyte meiosis recovery. 14 hours later, we detected and recorded amounts of oocytes transformed into metaphase of meiosis II (MII) in order to achieve information about the impact of the anti-CENP-C antibody on oocyte maturation. To obtain MII oocytes *in vivo*, 10 IU HCG were injected intraperitoneally into mice 48 hours after intraperitoneal injection of 10 IU pregnant mare serum gonadotropin (PMSG). MII oocytes were obtained 12-14 hours after HCG injection and used to test spindle morphology and chromosome alignment.

### 2.5. Immunofluorescence and Fluorescence Microscopy

#### 2.5.1. Detection of the Intracellular Anti-CENP-C Antibody

Oocytes were fixed with 4% paraformaldehyde for 30 min and then treated in 0.5% Triton X-100 for 20 min. After being blocked in 1% BSA in PBS for 1 h, samples were incubated with the secondary FITC-conjugated goat anti-mouse antibody for 2 hours at room temperature. Nuclear status was stained with DAPI (blue) for 10 min. After being briefly washed in PBS, oocytes were mounted on glass slides in a drop of the antifade medium and then examined with a fluorescence microscope (Axio Imager Z1, Zeiss, Oberkochen, Germany).

#### 2.5.2. Detection of Spindle Morphology and Chromosome Alignment of Mature Oocytes

Oocytes were fixed with 4% paraformaldehyde for 30 min and then treated in 0.5% Triton X-100 for 20 min. After being blocked in 1% BSA in PBS for 1 h, samples were incubated overnight at 4°C with the primary antibody: anti-tubulin antibody. After three washes for 10 minutes each, the oocytes were labeled with the secondary FITC-conjugated goat anti-mouse antibody for 1 hour at room temperature. Nuclear status was stained with DAPI (blue) for 10 min. After being briefly washed in PBS, oocytes were mounted on glass slides in a drop of the antifade medium and then examined with a fluorescence microscope (Axio Imager Z1, Zeiss, Oberkochen, Germany).

### 2.6. Western Blotting

Oocytes at the GV and GVBD stages were lysed using the Radioimmunoprecipitation Assay (RIPA) buffer (Fermentas, Waltham, MA, USA). Protein concentrations of the extracts were measured with a bicinchoninic acid assay (Beyotime, Shanghai, China). 20 *μ*g cell proteins were applied to 10% SDS-polyacrylamide gel. After electrophoresis, the proteins were transferred to polyvinyl difluoride membranes (Bio-Rad, Hercules, CA, USA). The membranes were incubated at 4°C overnight with the primary antibodies against CENP-C (ab193666, Abcam) or GAPDH (5174, CST). Then, the membranes were incubated with HRP-conjugated secondary antibodies for 1 h at room temperature and finally visualized using enhanced chemiluminescence reagents (Pierce, Waltham, MA, USA). The bands were analyzed by ImageJ software. The quantitative data were obtained by normalizing to GAPDH signals.

### 2.7. Statistical Analysis

Statistical analysis was performed with SPSS and GraphPad Prism (GraphPad Software). Data were representative of at least three independent experiments unless otherwise specified. Data were expressed as mean ± SEM from 3 independent experiments. The significance of differences between groups was analyzed by Student's *t*-test and the chi-squared test; *P* values < 0.05 were considered statistically significant.

## 3. Results

### 3.1. Serum CENP-C Antibody Detection in Mice

**Figure 1:**
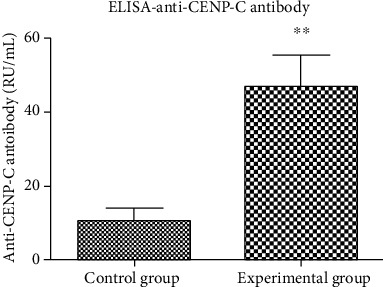
CENP-C antibody development in mouse serum. Concentration of the serum anti-CENP-C antibody in mice between the control group and the experimental group. ^∗^*P* < 0.05, ^∗∗^*P* < 0.01.

Whole blood samples of mice immunized with human CENP-C protein and of mice injected with CFA were collected on the oocyte pick-up day from mouse eyes. Mouse serum was obtained through centrifugation and was used to detect the CENP-C antibody level. The serum CENP-C antibody was quantitatively detected by the ELISA kit. The quantitative concentration of the serum CENP-C antibody in mice was higher in the experimental group injected with CENP-C protein. The value of the serum CENP-C antibody less than 20 RU/mL means negative according to the kit instruction. That is to say, the CENP-C antibody was only developed in the serum of mice in the experimental group (Figure 1).

### 3.2. Cellular Existence of the CENP-C Antibody in Oocytes

**Figure 2:**
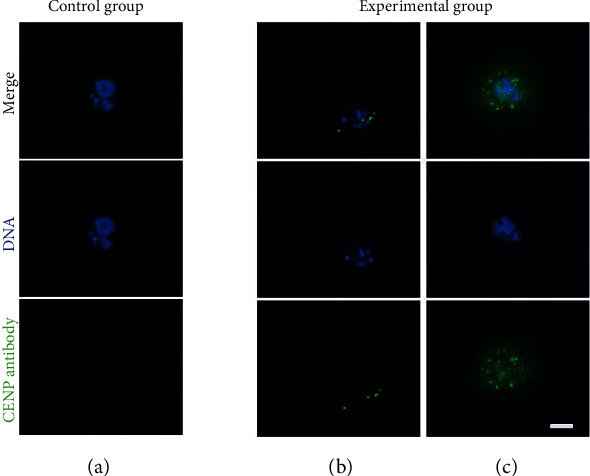
Cellular existence of the CENP-C antibody in mouse oocytes. Mouse oocytes from the control group and experimental group were immunolabeled with FITC-conjugated goat anti-mouse IgG for the CENP-C antibody (green) and counterstained with DAPI for chromosomes (blue). We detected whether the anti-CENP-C antibody was in oocytes at the GV stage and at the GVBD stage to ensure the antibody's entrance into the cell before metaphase. The CENP-C antibody found in GV oocytes (b) and GVBD oocytes (c) from the experimental group. The CENP-C antibody was not detected in GV oocytes from the control group. Images were captured using a 20x objective lens and 10x eyepiece. Scale bar: 20 *μ*m.

As we have just detected the CENP-C antibody in serum of experimental group mice, we wanted to investigate whether the anticentromere-C antibody existed in oocytes. Mouse oocytes from the experimental group and control group were immunolabeled with FITC-conjugated goat anti-mouse IgG for the CENP-C antibody and counterstained with DAPI for chromosomes. The CENP-C antibody was only found in oocytes of experimental group mice. The CENP-C antibody was not detected in oocytes of mice injected with CFA in the control group (Figure 2).

### 3.3. CENP-C Protein Was Decreased in Oocytes Obtained from the Experimental Group

**Figure 3:**
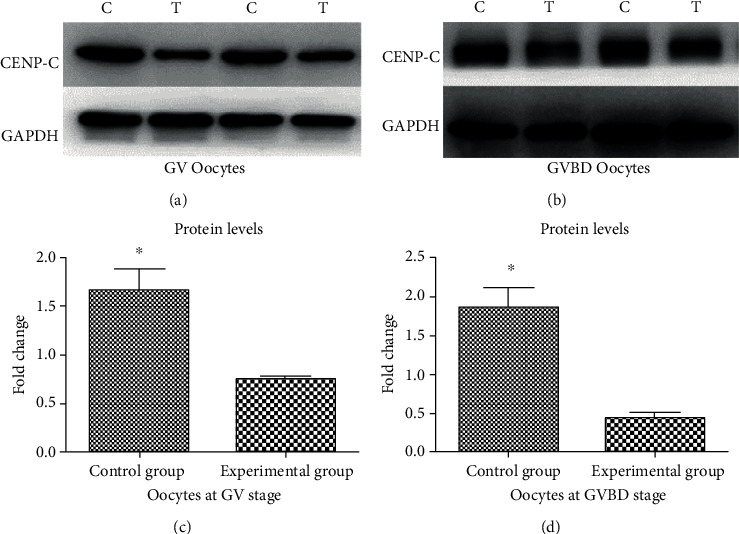
CENP-C protein in oocytes. Western blotting of CENP-C protein in total protein extracted from GV oocytes (a) and oocytes at the GVBD stage (b). The membranes were cut prior to exposure so that only the portion of gel containing desired bands would be visualized. C: control group; T: experimental group. Quantification of CENP-C protein in oocytes at the GV stage (c) and GVBD stage (d). ^∗^*P* < 0.05, ^∗∗^*P* < 0.01.

As the CENP-C antibody was detected in oocytes of mice in the experimental group, we wondered whether CENP-C protein declined in oocytes from the experimental group. CENP-C protein in the total protein extracted from oocytes in the experimental group was lower than CENP-C protein extracted from oocytes in the control group at the germinal vesicle (GV) stage by western blotting. CENP-C protein extracted from oocytes in the experimental group was lower than CENP-C protein extracted from oocytes in the control group at the germinal vesicle breakdown (GVBD) stage. The CENP-C antibody binding to CENP-C protein causes CENP-C protein to decrease after oocytes are lysed (Figure 3).

### 3.4. CENP-C Antibody Affected Mature Progression of Mouse Oocytes

**Figure 4:**
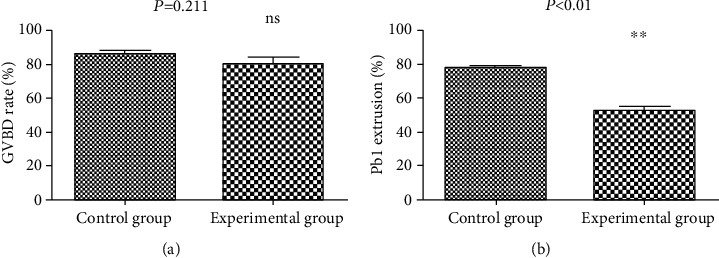
Effects of the CENP-C antibody on oocyte maturation. Oocytes during germinal vesicle breakdown were detected after 3 hours of culture from GV oocytes. Oocytes during first polar body (Pb1) extrusion were observed after 14 hours of *in vitro* maturation of GV oocytes. (a) Quantitative analysis of GVBD in the control group and experimental group (80.23 ± 3.71% vs. 86.27 ± 1.62% control, *P* = 0.211). (b) Quantitative analysis of Pb1 extrusion in the control group and experimental group (52.73 ± 2.03% vs. 77.90 ± 1.06% control, *P* < 0.01). Data were expressed as mean ± SEM from 3 independent experiments. ^∗∗^*P* < 0.01.

The process of oocyte maturation included germinal vesicle breakdown (GVBD), spindle assembly and chromosome alignment at the equator plate in metaphase of meiosis I (MI), first polar body (Pb1) extrusion, and oocyte stagnation in metaphase of meiosis II (MII) waiting for fertilization. Our results showed that the CENP-C antibody had no effect on meiotic resumption, as evidenced by the similar GVBD rate of oocytes after 3 hours of culture from GV oocytes between the experimental group and the control group (80.23 ± 3.71% vs. 86.27 ± 1.62% control, *P* = 0.211; Figure 4(a)). In contrast, the ratio of Pb1 extrusion of oocytes was significantly decreased in the experimental group after 14 hours of *in vitro* maturation from GV oocytes (52.73 ± 2.03% vs. 77.90 ± 1.06% control, *P* < 0.01; Figure 4(b)), implying the detrimental role of the CENP-C antibody in oocyte maturation. Total experimental data obtained from 3 independent experiments were collected and statistically analyzed. The results also showed that the CENP-C antibody did not affect the recovery of oocyte meiosis but affected oocyte maturation (Tables 1 and 2).

### 3.5. CENP-C Antibody Impaired Spindle Organization and Chromosome Alignment in Oocytes

**Figure 5:**
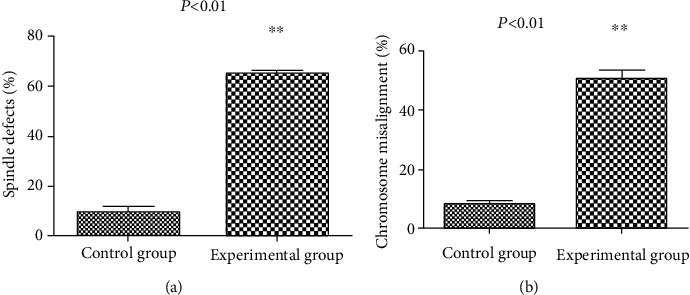
(a) Quantification of control and experimental oocytes with abnormal spindles (64.67 ± 1.16% vs. 9.27 ± 2.28% control, *P* < 0.01). (b) Quantitative analysis of control and experimental oocytes with chromosome misalignment (50.80 ± 2.40% vs. 8.30 ± 1.16% control, *P* < 0.01). Data were expressed as mean ± SEM from 3 independent experiments. ^∗∗^*P* < 0.01.

**Figure 6:**
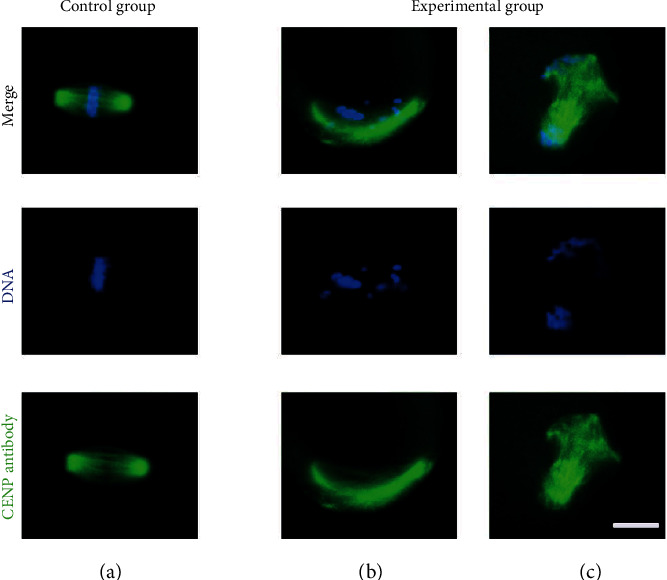
Effects of the CENP-C antibody on spindle assembly and chromosome alignment in MII oocytes. Control and experimental oocytes were stained with the tubulin antibody to visualize spindles (green) and counterstained with DAPI to visualize chromosomes (blue). Control oocytes present a bipolar barrel-shaped spindle and well-aligned chromosomes on the metaphase equator (a), whereas spindle defects and chromosome misalignment were observed in experimental oocytes (b, c). Images were captured using a 40x objective lens and 10x eyepiece. Scale bar: 20 *μ*m.

As the CENP-C antibody existed in oocytes derived from the experimental group and affected the mature progression of oocytes, we asked whether the CENP-C antibody impaired the assembly of the meiotic apparatus in oocytes. To gain insight into this issue, mouse oocytes in MII retrieved directly from mice *in vivo* in the experimental group and control group were immunolabeled with the anti-tubulin antibody for spindles and counterstained with DAPI for chromosomes. We found a higher percentage of spindle defects and chromosome congression failure in the experimental group (spindle defects: 64.67 ± 1.16% vs. 9.27 ± 2.28% control; chromosome misalignment: 50.80 ± 2.40% vs. 8.30 ± 1.16% control) (Figure 5). Spindle defects included spindle elongation and spindle irregularity. One or several chromosomes detached from the metaphase plate and drifted into the cytoplasm were chromosome congression failures (Figures 6(b) and 6(c)). Normal spindles are barrel-shaped, short, and nearly round at metaphase. Chromosomes are aligned at the equatorial plate plane orderly. By striking contrast, oocytes in the control group at the metaphase stage usually showed a typical barrel-shaped spindle and well-aligned chromosomes at the equator plate (Figure 6(a)). Total experimental data obtained from 3 independent experiments were collected and statistically analyzed. The results also showed that the CENP-C antibody could cause higher spindle defects and chromosome misalignment in the oocytes of the experimental group (Tables 3 and 4).

## 4. Discussion

Reproductive autoimmune failure syndrome referred to the antibodies that could be detected in women's body fluids who had no clinical symptoms of autoimmune diseases. This abnormal immune status could cause reproductive failures such as infertility and abortion. The abnormal level of antibodies and IVF/ICSI failure should be considered clinical symptoms of autoimmune imbalance even if clinical manifestations of autoimmune diseases did not appear [12]. All sterile women with serum positive ACA in our reproductive medicine center have no clinical signs of autoimmune diseases. Therefore, mice immunized with centromere protein were used for investigating the role of the centromere antibody in oocyte meiosis, which possessed high clinical application value for ACA-positive women. ACA, among types of the antinuclear antibody, were relevant to adverse reproductive events, including the decreased rate of mature oocytes and the lower rate of embryo cleavage [1, 6].

There were research studies that showed that ANA, such as the anti-DNA antibody and anti-ribosomal P protein antibody, could penetrate into living cells and interact with their intracellular targets [13–15]. These studies from several laboratories contradicted the prevailing immunologic dogma that cell interiors were inaccessible to antibodies. The development of embryos cocultured with IgG, which was retrieved from the serum of ANA-positive patients, was severely damaged [16]. In our experiment, we also proved that the anti-CENP-C antibody could penetrate oocytes. Human oocyte meiosis began in the fetal period and stagnated in prophase of meiosis I. The typical character in prophase of meiosis I was a huge nucleus called the germinal vesicle (GV) in oocytes. Oocyte meiosis recovery was marked by germinal vesicle breakdown (GVBD) from puberty under the effect of hormones. Oocytes stagnated once again in metaphase of meiosis II until fertilization. The rate of GVBD was not significantly different between the experimental group and the control group, and the rate of the first polar body extrusion was higher in the control group of our experiment. We concluded that the anti-CENP-C antibody did not affect the oocyte meiosis recovery but made mature oocytes decrease.

Kinetochores, including more than 100 proteins, were a compact structure in centromeres. The functions of kinetochores include microtubule capture, spindle stabilization, metaphase chromosome congression, and chromosome movement [11]. Kinetochores undergo a program of morphogenesis at different stages of the cell cycle. During interphase, kinetochores could not be distinguished from the surrounding centromeric heterochromatin. At prophase, the structure is first visible as a fuzzy ball at the surface of the centromeric chromatin. This differentiates into characteristic trilaminar disks during metaphase [7]. The outer kinetochore, including Mis12 complex, Knl1 complex, and Ndc80 complex, was in charge of stable kinetochore-microtubule connection and recruitment of the spindle assembly checkpoint (SAC), which is crucial for cell division. SAC is an elementary self-monitoring system in eukaryotic cell division. SAC senses kinetochores that were not unattached with spindles, then forms the “wait anaphase” signal, which prolongs cell premetaphase time in cell division. At the prolonged time, specific repaired pathways, which consist of protein-protein interaction, initiate and prevent the appearance of premature chromosome separation and aneuploidy [17, 18]. Even a single kinetochore that does not bind to a microtubule could activate SAC [19]. Hence, the destruction of kinetochore as a platform for kinetochore-microtubule (KT-MT) attachment and SAC activity could cause abnormal cell division. Both structure and function between centromere proteins and kinetochore proteins are interactional and interdependent. There are two main patterns to explain their interplay. First, the C-terminal and central regions of CENP-C interact with CENP-A, and the N-terminal of CENP-C binds to Mis12, which suggests CENP-C links the centromeric chromatin with the outer kinetochore. Second, the CENP-T/W/S/X compound connects centromere DNA; in addition, CENP-T directly binds to the outer kinetochore Ndc80 complex [20]. Kinetochore-microtubule (KT-MT) attachment defects and chromosome separation errors would occur if the Ndc80 complex is damaged [21]. Therefore, some common molecules form the structure of centromere proteins and kinetochore proteins. Abnormal CENP could destroy the construction and function of kinetochore proteins, which would lead to aberrant cell division. Acquisition of haploid eggs in oocyte meiosis results from correct chromosome segregation. Aberrant centromere protein could destroy the structure and function of kinetochores, which results in abnormal mitosis and meiosis.

CENP-A/B/C constitute basic CENP complexes. At present, CENP-H/M/N/T/U(50) are also found in CENP complexes. These CENP components form the Constitutive Centromere-Associated Network (CCAN) [22]. At the hub of kinetochore assembly, CENP-C is expressed in the active centromere of diplotene chromosomes. CENP-C links the centromeric chromatin with the outer kinetochore, and CENP-C's depletion causes the destruction of the outer kinetochore, which is essential to cell division [23, 24]. The importance of CENP-C in cell division was also investigated in some studies. The following appearances would occur after the CENP-C antibody was injected into interphase of HeLa cells: the cell division cycle arrested at metaphase, the number and size of kinetochores reduced, remaining kinetochores no longer connected with spindle microtubules, and chromosome congression disrupted [25]. Mouse embryos with CENP-C gene knockout only lived for 3.5 days as chromosome segregation was wrong [26]. The cell cycle of chicken DT-40 cells with absent CENP-C showed premetaphase arrest and cell apoptosis [27]. Therefore, CENP-C was indispensable for chromosome segregation and cell proliferation. ACA were first found in the serum of CREST syndrome patients, which mainly included CENP-A, CENP-B, CENP-C, and CENP-D antibodies [28]. Impacts of ACA injected into cells on mitosis and meiosis at different cell cycle times were different. ACA injected into mitotic cells at interphase resulted in damaged kinetochores and unstable kinetochore-microtubule (KT-MT) attachment [25, 29, 30]. ACA injected into oocytes at interphase caused chromosome congression failure in the first and second meiotic divisions, while microtubule capture, spindle formation, and chromosome segregation at anaphase appeared unaffected [31]. When ACA were microinjected into cells at metaphase, the processes of mitosis and meiosis were normal. Chromosome alignment was dramatically influenced when ACA were introduced during the interphase or prometaphase stage [31]. Outer kinetochore assembly was sensitive to ACA 2 hours before mitosis, and inner kinetochore assembly was sensitive to ACA up until the beginning of prophase [30]. The earlier the ACA penetrate into cells, the greater the detrimental effects of ACA on cell division. We speculated that ACA should enter into cells before metaphase. The studies above offered some pieces of evidence for the significance of ACA on cell division *in vitro*, but these studies possessed certain limitations. As ACA include CENP-A, CENP-B, CENP-C, and CENP-D antibodies, we do not know which specific antibody plays a leading role in cell division. In our study, we detected that the anti-CENP-C antibody was in oocytes at the GV stage and at the GVBD stage to ensure the antibody's entrance into the cell before metaphase and investigated the influence of the CENP-C antibody developed *in vivo* by the method of autoimmunity on oocyte meiosis. In this study, we found that the CENP-C antibody could be detected in oocytes of mice immunized with CENP-C protein and could adversely impact oocyte maturation, spindle organization, and chromosome alignment. Proper chromosome segregation assures the acquisition of accurate and complete genetic material of haploid eggs divided from diploid cells. Normal spindle morphology and accurate metaphase chromosome alignment are two main procedures to ensure correct chromosome separation and acquisition of haploid eggs [32]. Spindle defects and metaphase chromosome misalignment could cause aneuploidy, which are the reasons for infertility, abortion, and fetal birth defects. Deficient spindle structure and chromosome misalignment in metaphase of meiosis II could result in aneuploidy oocytes. Fertilization of these abnormal eggs is the main cause of infertility, recurrent spontaneous abortion, and subsequent embryonic development defects. Maybe this is the reason for adverse reproductive outcomes in ACA-positive patients. Antibodies in CREST serum are rarely monospecific for a single centromere antibody, and so it might be premature to conclude that only the CENP-C antibody could impact oocyte meiosis. But CENP-C is required for kinetochore assembly and microtubule attachment stabilization. The CENP-C antibody may play a more important role in cell division. This study revealed that immunologic factors could affect oocyte meiosis and cause abnormal spindles and chromosome misalignment in oocytes, which could provide some new thoughts and clues for improving reproductive outcomes of ACA-positive women with no clinical symptoms of autoimmune diseases, decreasing fetal birth defects, and increasing assisted reproductive success rates. Further experiments are required to validate these conclusions.

## 5. Conclusion

We found that the CENP-C antibody produced by the autoimmune method in mice could penetrate oocytes and cause oocyte maturation and spindle defects and penetrate chromosomes that were detached from the metaphase plate and scattered in the cytoplasm in metaphase, which may be the main mechanism of adverse reproductive outcomes for anticentromere antibody-positive women who have no clinical symptoms of any autoimmune diseases.

## Figures and Tables

**Table 1:** Comparison of GVBD between the experimental group and the control group.

GVBD	Experimental group (*n* = 131)	Control group (*n* = 145)	Total
Happened	105 (80.2%)	125 (86.2%)	230
Unhappened	26 (19.8%)	20 (13.8%)	46
Total	131 (100%)	145 (100%)	276

*χ* ^2^ value: 1.816. *P* value: 0.178. *P* < 0.05 was considered statistically significant.

**Table 2:** Comparison of polar body discharge between the experimental group and the control group.

Polar body discharge	Experimental group (*n* = 131)	Control group (*n* = 145)	Total
Discharge	69 (52.7%)	113 (77.9%)	182
Undischarge	62 (47.3%)	32 (22.1%)	94
Total	131 (100%)	145 (100%)	276

*χ* ^2^ value: 19.552. *P* < 0.01. *P* < 0.05 was considered statistically significant.

**Table 3:** Comparison of the spindle shape between the experimental group and the control group.

Spindle shape	Experimental group (*n* = 147)	Control group (*n* = 150)	Total
Normal	52 (35.4%)	136 (90.7%)	188
Abnormal	95 (64.6%)	14 (9.3%)	109
Total	147 (100%)	150 (100%)	297

*χ* ^2^: 97.704. *P* < 0.01. *P* < 0.05 was considered statistically significant.

**Table 4:** Comparison of chromosome alignment between the experimental group and the control group.

Chromosome alignment	Experimental group (*n* = 130)	Control group (*n* = 144)	Total
Normal	64 (49.2%)	132 (91.7%)	196
Abnormal	66 (50.8%)	12 (8.3%)	78
Total	130 (100%)	144 (100%)	274

*χ* ^2^: 60.419. *P* < 0.01. *P* < 0.05 was considered statistically significant.

## Data Availability

All data are available in our manuscript.
